# Perceptions of Parenting Practices and Psychological Variables of Elite and Sub-Elite Youth Athletes

**DOI:** 10.3389/fpsyg.2019.01495

**Published:** 2019-06-28

**Authors:** Pedro Teques, Luís Calmeiro, António Rosado, Carlos Silva, Sidónio Serpa

**Affiliations:** ^1^Polytechnic Institute of Maia, Maia, Portugal; ^2^CIPER, Interdisciplinary Center for the Study of Human Performance, Lisbon, Portugal; ^3^School of Social and Health Sciences, Abertay University, Dundee, United Kingdom; ^4^Institute of Environmental Health, Faculty of Medicine, University of Lisbon, Lisbon, Portugal; ^5^Faculty of Human Kinetics, University of Lisbon, Lisbon, Portugal; ^6^CIEQV, Sport Sciences School of Rio Maior, Polytechnic Institute of Santarém, Santarém, Portugal; ^7^Lusophone University of Humanities and Technologies, Lisbon, Portugal

**Keywords:** intrinsic motivation, parental roles, self-efficacy, self-regulation, structural equation modeling, youth sport

## Abstract

Drawing from the model of parental involvement in sport, the overall purpose was to examine the associations of perceptions of parenting practices (encouragement, reinforcement, instruction, and role modeling) and athletes’ psychological variables (self-efficacy, social self-efficacy, self-regulation, and intrinsic motivation) of elite and sub-elite youth athletes. Participants were elite (*n* = 210) and sub-elite (*n* = 635) athletes aged between 14 and 18 years (*M*_*age*_ = 16.58, *SD* = 1.33). Structural equation modeling analysis revealed that young elite athletes’ perceptions of sport-related parenting practices are associated with their psychological skills and performance level in sport. Specifically, in comparison with their sub-elite peers, perceptions of parental encouragement had a significantly different strong effect on intrinsic motivation. Moreover, perceptions of parental modeling revealed different effects on performance level, as well as on intrinsic motivation, and self-regulation. These perceptions of parenting practices may promote a positive learning environment, resulting in an increased likelihood of achieving a high level of sport performance in comparison with their sub-elite peers.

## Introduction

Early achievement of elite in sport is influenced by the type of experiences that young athletes have throughout their development, including the psychosocial relationships they establish with their parents (Côté, 1999). Although researchers have tested the influence of parents’ behaviors on young athletes’ psychological variables in sport (e.g., Babkes and Weiss, 1999; Fredricks and Eccles, 2005), research has generated few empirical data demonstrating how parenting practices influence young athletes’ attainment of high levels of performance in sport. In fact, theoretical frameworks that guided research on parents’ influences on differential child outcomes in sport and physical activity, such as the Eccles’ model of parental influence on children’s motivation and achievement (e.g., Fredricks and Eccles, 2004) do not specify the characteristics of parents’ involvement and support in competitive sport situations (Holt et al., 2008).

To further understand parental influence in youth sport, Teques and Serpa (2009, 2013) and Teques et al. (2015, 2018) adapted the model of parental involvement in sport, originally developed by Walker et al. (2005). In the present study, we use this model as a framework to specifically examine the associations between parenting practices and psychological skills of elite and sub-elite youth athletes.

### Theoretical Model

The model of parental involvement in sport (Teques and Serpa, 2009, 2013; Teques et al., 2015, 2018) was developed to explain why parents get involved in their children’s sport, what type of behaviors they display during their involvement, and how their involvement influences young athletes’ psychological variables in sport. Recent empirical studies (Teques et al., 2015, 2018) have shown parents’ involvement decisions were primarily influenced by (a) a personal construction of a sport specific parental role, (b) the outcomes they expect will follow from their actions to support young athletes development, (c) their perceptions of invitations from the athlete and the coach to be involved, (d) their knowledge and skills about the athlete’s sport, (e) and their assessment of time and energy to support the athlete’s needs (Teques et al., 2015). During their involvement, parents’ reported behaviors (i.e., role modeling, reinforcement, encouragement and technical instruction) are associated with the athletes’ psychological variables conducive to achievement in sport (i.e., self-efficacy, social self-efficacy with their coach, self-regulation, intrinsic motivation) via athletes’ perceptions of parents’ behaviors (Teques et al., 2018). The focus of this study was on how young athletes’ perceptions of their parents’ behaviors and athletes’ psychological variables are associated with their level of performance in sport.

### Perceptions of Parents’ Behaviors and Elite Performance in Youth Sport

Athletes’ perceptions of the dimensions of parental involvement practices have been consistently associated with achieving an elite status in sport (e.g., Wolfenden and Holt, 2005; Clarke et al., 2016; Knight et al., 2016). For example, Holt and Dunn (2004) conducted interviews to explore psychological skills among young elite soccer players. Data analysis showed the importance of parents in providing encouragement to support adolescents cope with stressors associated to elite youth sport.

Parents’ modeling behaviors have the potential to influence children’s behaviors through observational learning (Bandura, 1997). In talent development studies, children’s perceptions of parents as models of hard work who set high standards of performance were reported to influence their achievement in sport. Parents also influence their children’s participation in sport through the provision of directive behaviors, such as technical instructions. Specifically, Holt et al. (2008) suggested that parents’ instruction behaviors during games accounted for more than one third of the recorded parental comments. In addition, Wuerth et al. (2004) found that athletes who progressed into a higher career stage, reported much more directive behavior from their parents (e.g., showing children how to improve and pushing them to train harder are embedded in praise for trying hard, warmth and understanding) than those athletes who stayed in the same career stage.

### Psychological Variables and High Level of Performance in Sport

As several researchers in sport parenting literature have suggested (e.g., Babkes and Weiss, 1999; Woolger and Power, 2000) athletes’ development of psychological attributes may mediate the relationship between parents’ behaviors and young athletes’ experiences in sport. Therefore, the theoretical model of parental involvement in sport (Walker et al., 2005; Teques and Serpa, 2009, 2013; Teques et al., 2015, 2018) identifies four main athletes’ psychological attributes related to achievement of high level of performance which can be influenced by parental behaviors. These attributes consist of self-efficacy, social efficacy to relate with the coach, intrinsic motivation, and self-regulatory strategies.

In conceptualizing intrinsic motivation, Ryan and Deci (2006) proposed that people are motivated by innate needs for self-determination. For example, Mallett and Hanrahan (2004) reported that self-determined behaviors of elite athletes comprise enjoyment of sport tasks characterized by an orientation toward mastery, persistence and strong desire to achieve personal goals. The sense of goal accomplishment is associated with self-determined forms of motivation.

According to Bandura (1997), self-efficacy beliefs determine the goals individuals set for themselves, how much effort they exert, and their resilience to failure. Previous studies focusing on the differences of self-efficacy beliefs between elite and non-elite athletes reported conflicting results. For instance, Wilhelm et al. (2013) found that elite handball athletes achieve a higher perceived efficacy in comparison with non-elite athletes, whereas Toering et al. (2009) found no significant differences between groups. It has been suggested that parental encouragement and positive role modeling were related to adolescents’ self-efficacy in physical activity (de Farias Júnior et al., 2014).

Another psychological attribute conducive to achievement of a high level of performance is self-regulation. Adolescent elite athletes distinguish themselves from their non-elite and sub-elite peers by their superior self-regulatory skills, underscoring the importance of self-reflection. These findings support the evidence that athletes who reflect upon their actions are likely to know when they make errors, which enables these athletes to learn (Toering et al., 2009).

Finally, although studies on perceptions of parents’ social efficacy to relate with the coach were not found, the relevance of such conceptualization is based on studies developed in the academic domain that demonstrate an association between parents’ social efficacy to interact with teachers and students’ academic performance (Patrick et al., 2007). Similarly, studies in the sport domain support the influence of parents in the relationship between athletes and coaches (Jowett and Timson-Katchis, 2005). For example, Jowett and Timson-Katchis (2005) interviewed coach-athlete-parent links and showed that parents provide a variety of information and emotional support that are susceptible of influencing the value of the relationship between coach and athlete. Also, Averill and Power (1995) found that children who observe their parents’ directive behaviors (i.e., they directly interfere with the coach’s instructions, undermine the coach’s authority, and annoy the coach with concerns for special treatment) showed low levels of cooperation with the coach, suggesting that high levels of parental involvement undermine the coach-athlete relationship. Hence, it is suggested that studies should aim to explain how athletes’ perceptions of parents’ behaviors interact with the sense of efficacy for relating with their coach.

### Aim and Hypothesis

**FIGURE 1:**
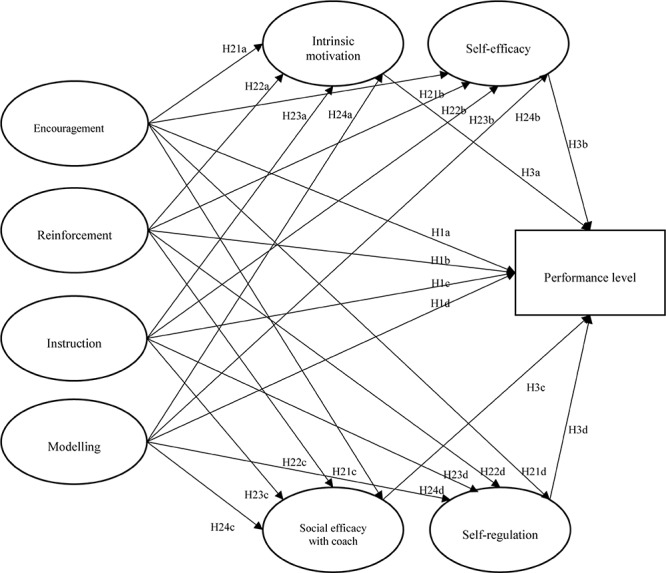
Hypothesized model.

The main purpose of this study was to investigate the associations of perceptions of parenting practices and athletes’ psychological attributes in elite and sub-elite participants. In particular, the focus of this study was on the differences between two groups of youth athletes: (a) elite athletes who belong to a national squad (i.e., selection of the best players of that sport who represent the country at international events), and (b) sub-elite athletes who compete at regional level, but they were never selected for national teams. On the basis of the model of parental involvement in sport, as shown in Figure 1, this study hypothesizes that perceptions of parenting practices, such as (a) encouragement, (b) reinforcement, (c) role modeling, and (d) technical instruction are directly linked with athletes’ playing level (Hypothesis 1). Second, it is hypothesized that perceptions of parents’ behaviors concerning (a) encouragement, (b) reinforcement, (c) role modeling, and (d) technical instruction will be significantly associated with young athletes’ self-efficacy, social efficacy to relate with the coach, intrinsic motivation, and self-regulatory strategies (Hypothesis 2). Third, this study hypothesizes that young athletes’ psychological variables, such as (a) self-efficacy, (b) social efficacy to relate with the coach, (c) intrinsic motivation, and (d) self-regulatory strategies, are associated with athletes’ playing level (Hypothesis 3).

## Materials and Methods

### Participants

Eight hundred and eighty-one young athletes (nboys = 689, ngirls = 192) aged between 14 and 18 years participated in the study. The sample comprised participants from the north, center, and south of the littoral regions of Portugal. Participants played a variety of team sports: soccer (44.6%), basketball (21.9%), handball (26.5%), and volleyball (7.0%). Because of the goal of the present study, only participants who identified the father and/or the mother as the person in their family who accompany them in their sport activities were eligible. Thus, data from 36 participants (nboys = 28, ngirls = 8) were withdrawn and the analyses were based on the remaining 845 athletes. The final sample included 210 elite and 635 sub-elite athletes aged between 14 and 18 years (Mage = 16.58, SD = 1.33). Table 1 presents the participants’ demographic information. Elite athletes were classified as those who participated in national teams. Sub-elite athletes competed at regional level. Athletes were representative of four age groups: Under-15 (30.0%), Under-16 (36%), Under-17 (21.3%), and Under-18 (12.7%). On average, participants practiced 5.3 h per week and had 9.2 years of experience in their current sport.

**TABLE 1:** Characteristics of participants.

	**Elite (*n* = 210)**	**Sub-elite (*n* = 635)**	**Total (*n* = 845)**
Age (*M*, *SD*)	(16.77, 1.34)	(16.23, 1.14)	(16.58, 1.33)
**Age groups**			
Under-15	66	188	254
Under-16	81	223	304
Under-17	45	135	180
Under-18	18	89	107
**Gender**			
Boys	132	529	661
Girls	78	106	184
**Sport**			
Basketball	58	127	185
Handball	46	178	224
Soccer	88	289	377
Volleyball	18	41	59
Training (hours p/week)	(7.1, 1.2)	(4.9, 1.8)	(5.3, 2.2)
Years of competitive experience	(9.8, 2.3)	(9.1, 2.7)	(9.2, 2.4)

### Procedure

This study was approved by the Ethical Committee of the Faculty of Human Kinetics of the University of Lisbon. Club directors and coaches from 23 sporting academies or clubs and four sport Federations authorized the researchers to directly contact athletes for participation. Parental consent forms and information sheets were given to all participants, which were returned when signed by the legal guardians (return rate of 92%). Prior to the administration of the questionnaires, it was made clear to all athletes that completion of the questionnaire was voluntary and that all responses would be kept confidential. Athletes’ completed the paper and pencil questionnaires during team training camps, or before regular training sessions. A member of the research team collected the data at each sports club and answered any questions during the data collection. Questionnaires were handed to a research team member immediately upon completion to avoid coaches or other respondents from having the opportunity to examine athletes’ questionnaires. Participants took about 15–20 min to complete the questionnaires pack.

### Measures

Psychometric scales were used to assess perceptions of parents’ behaviors and athletes’ psychological variables. All measures employed a four-point Likert-type response scale, ranging from 1 (not at all true) through 4 (very true). All items are presented in the Table 2.

**TABLE 2:** Factor loadings, composite reliability (C.R.), and average variance extracted (AVE).

	**Elite**			**Sub-elite**		
**Construct/Items**	**Loading**	**C.R.**	**AVE**	**Loading**	**C.R.**	**AVE**
**Encouragement:** *The person in my family who accompanied me in my sport encourages me…*						
To strive in practices and competitions	0.751	0.84	0.57	0.811	0.84	0.57
To believe that I can do well in (…)	0.826			0.836		
To stick with my problems until I solves it	0.637			0.656		
To believe that I can learn new things (…)	0.793			0.724		
**Reinforcement:** *The person in my family who accompanied me in my sport shows me that he/she likes it when I…*						
Put maximum effort in practices (…)	0.871	0.88	0.65	0.846	0.88	0.66
Have a good performance	0.870			0.810		
Have a good attitude in practices and competitions	0.759			0.826		
Try hard	0.724			0.771		
**Instruction:** *The person in my family who accompanied me in my sport tells me…*						
Instructions during competitions	0.768	0.87	0.62	0.783	0.79	0.68
How to do things before the game	0.829			0.851		
How to do to be better	0.809			0.873		
What I did wrong or right after the game	0.759			0.797		
**Modeling:** *The person in my family who accompanied me in my sport…*						
Does not give up in face of difficulties	0.744	0.77	0.53	0.630	0.71	0.44
Works hard to achieve things	0.626			0.628		
Gives importance to the effort to achieve (…)	0.673			0.621		
gives the best he/she can (…)	0.764			0.740		
**Intrinsic motivation**						
I feel a lot of personal satisfaction (…)	0.828	0.84	0.57	0.760	0.83	0.56
For the pleasure I feel while improving (…)	0.713			0.791		
For the satisfaction I experience while (…)	0.671			0.680		
For the pleasure that I feel while executing (…)	0.808			0.758		
**Social self-efficacy with coach**						
I can get along with most of my coaches	0.665	0.86	0.62	0.749	0.85	0.59
I can explain what I think to most of my coaches	0.827			0.743		
I can get my coaches to help me if I have (…)	0.857			0.805		
I can get my coaches to help me develop (…)	0.797			0.790		
**Self-efficacy**						
I can always manage to solve difficult (…)	0.705	0.79	0.49	0.689	0.80	0.51
I can solve most problems if I invest (…)	0.719			0.687		
I can remain calm wen facing difficulties (…)	0.645			0.717		
I can usually handle whatever comes my way	0.739			0.760		
**Self-regulation**						
I reappraise my experiences so I can learn (…)	0.608	0.83	0.56	0.721	0.80	0.50
I try to think about my strengths (…)	0.757			0.661		
I think about my actions to see whether I can (…)	0.819			0.695		
I try to think about how I can do things better (…)	0.797			0.757		

#### Perceptions of Parents’ Behaviors

Four types of athletes’ perceptions of parental involvement behaviors were examined (Teques et al., 2018): encouragement, reinforcement, instruction, and modeling. In order to assess perceptions of parents’ encouragement, parents’ explicit support to the young athlete’s activities in sport were measured. Items referring to parents’ expressed satisfaction when the child improves, learns new skills, and works hard in sport were included to address perceptions of parents’ reinforcement behaviors. The instruction scale included items to assess athletes’ perceptions of parents’ technical instructions before, during and after their sports competitions. The modeling scale was aimed to measure perceptions of parental behaviors as values of hard work that impacts young athletes’ sport experiences.

#### Psychological Variables

This study used four scales to assess athletes’ psychological variables in sport: self-efficacy, social efficacy to relate with the coach, intrinsic motivation, and self-regulatory strategies. The sport efficacy scale refers to athletes’ judgments of their capability to act in ways that will result in successful performance in their competitive sport. The measure of self-efficacy was adapted from the General Self-Efficacy Scale (Schwarzer and Jerusalem, 1995). The social self-efficacy scale with coach included items to assess athletes’ evaluations of their capacity to relate effectively with their coach. Thus, items from the Perceived Social Efficacy with Teacher (Patrick et al., 2007) were adapted to the sport context. The self-regulation strategies scale is based on a wide set of metacognitions and behaviors, such as self-reflection. Athletes self-evaluate the actions employed and the outcome achieved to improve next performance. Four items adopted from the Reflection subscale of the Self-regulation of Learning Self-report Scale (Toering et al., 2012) measured self-regulation. The intrinsic motivation scale focused on athlete’s interest in sport practice for the pleasure resultant from trying to exceed oneself or to improve one’s skills. Four items of the Intrinsic Motivation to Accomplish subscale of the Sport Motivation Scale were used (Pelletier et al., 1995).

#### Performance Level

Performance was defined as the level of participation attained by young athletes in their specific sport classified in two types: elite and sub-elite. Elite athletes were those who were selected in the last year for the national teams in their sports and have represented the country at international events. Sub-elite athletes were those who compete only at regional level in their sport and were never selected for national teams. Performance was coded according to youth athletes’ playing level in their sport: 0 = sub-elite and 1 = elite.

### Data Analysis

A two-step approach to maximum likelihood structural modeling were performed using IBM SPSS AMOS 23 (Analysis of Moment Structures; IBM Corp., Armonk, NY, United States). First, the measurement model was assessed conducting a confirmatory factor analysis (CFA). The internal consistency reliability estimates were evaluated through composite reliability (composite reliability ≥0.70; Hair et al., 2014). Convergent and discriminant validity were assessed to test construct validity. The average variance extracted (AVE) was estimated to evaluate convergent validity with values greater than 0.50 indicating convergent validity (Fornell and Larcker, 1981). The criterion to assume discriminant validity was that the AVE for each construct was larger than the inter-construct squared correlation (Fornell and Larcker, 1981). We followed recommendations from Hair et al. (2014) to assess the adequacy of the model through a variety of fit indices: CFI (comparative fit index) and TLI (Tucker Lewis index) >0.90, RMSEA (root mean square error of approximation) and SRMR (standardized root mean square residual) <0.08.

Subsequently, the structural model was performed to test hypothesis. Also, a multi-group analysis was conducted in order to identify differences on the path coefficients among models for the elite and sub-elite groups. Following Byrne’s (2010) suggestions, structural invariance between models was examined with chi-square (χ^2^) and CFI difference (ΔCFI) values. The premise of invariance will be accepted if χ^2^ is not statistically significant (*p* > 0.05); however, χ^2^ is permeable to sample size and consequently changes in the ΔCFI of greater than 0.01 will be considered. The invariance between elite and sub-elite groups was evaluated by sequentially comparing the unconstrained model with the constrained measurement weights model and the constrained structural weights model. The statistical significance of the structural weights was assumed when critical ratios (CR) for differences among parameters produced by AMOS showed values >1.96.

## Results

### Preliminary Analyses

A preliminary screen to the data was conducted to collect information about outliers, missing values, normality, and collinearity, as suggested by Hair et al. (2014). Missing values comprise 4.2% of cells in the original data, without any missing data patterns. Thus, missing data were imputed using AMOS’s regression procedure. Twelve multivariate (Mahalanobis distance = *p1* and *p2* < 0.001) and univariate (*z* < 3.00) outliers emerged (eight cases in the sub-elite and four cases in the elite sample). These cases were removed from subsequent analyzes. Mardia’s coefficient (38.74) exceeded minimum values for the multivariate normality. Hence, a Bollen-Stine bootstrap (B-S) on 2000 samples was used for subsequent analysis, as recommended by Nevitt and Hancock (2001). In addition, variance inflation factors were assessed to verify collinearity within all study variables, with values ranging from 1.12 (instruction) to 1.81 (encouragement), showing acceptable conditions to conduct regression analysis (Hair et al., 2014).

### Measurement Model

Table 2 shows means, standard deviations and squared correlations among all variables in both subsamples. The mean scores showed that youth elite athletes revealed higher intrinsic motivation to accomplish (*M* = 3.49, *SD* = 0.51) and sub-elite showed lower levels of perceived parental instruction (*M* = 2.43, *SD* = 0.86). All variables are close to or exceeded the level greater than 0.50 of AVE for convergent validity, ranging from 0.49 to 0.65 (elite), and from 0.44 to 0.68 (sub-elite). AVE estimates for each construct were larger than the inter-construct squared correlation, supporting the discriminant validity of all variables. Additionally, the reliability coefficients were greater than 0.70 (Hair et al., 2014) in both models (Table 3).

**TABLE 3:** Means (M), standard deviations (SD), and squared correlations.

			**Correlation matrix**						
**Construct**	***M***	***SD***	**1**	**2**	**3**	**4**	**5**	**6**	**7**
**Elite**									
(1) Encouragement	3.29	0.57	1.00						
(2) Reinforcement	3.41	0.59	0.31**	1.00					
(3) Instruction	2.49	0.81	0.04**	0.06**	1.00				
(4) Modeling	3.41	0.51	0.22**	0.18**	0.05**	1.00			
(5) Intrinsic mot.	3.49	0.51	0.21**	0.12**	−0.03*	0.18**	1.00		
(6) Social efficacy	3.30	0.54	0.18**	0.05**	−0.08*	0.08**	0.15**	1.00	
(7) Self-efficacy	3.29	0.50	0.15**	0.18**	−0.02*	0.12**	0.16**	0.20**	1.00
(8) Self-regulation	3.38	0.55	0.15**	0.12**	0.01	0.12**	0.26**	0.18**	0.29**
**Sub-elite**									
(1) Encouragement	3.29	0.59	1.00						
(2) Reinforcement	3.36	0.61	0.30**	1.00					
(3) Instruction	2.43	0.86	0.02**	0.04**	1.00				
(4) Modeling	3.25	0.55	0.24**	0.23**	0.01*	1.00			
(5) Intrinsic mot.	3.37	0.50	0.13**	0.10**	−0.01*	0.06**	1.00		
(6) Social efficacy	3.34	0.57	0.06**	0.07**	−0.01*	0.07**	0.15**	1.00	
(7) Self-efficacy	3.26	0.51	0.09**	0.11**	−0.00	0.04**	0.11**	0.18**	1.00
(8) Self-regulation	3.25	0.53	0.13**	0.12**	0.00	0.07**	0.19**	0.22**	0.33**

No squared correlations failed the AVE test of discriminant validity. *p < 0.05, **p < 0.01.

The measurement model for youth elite [χ2(436) = 675.32, *p* < 0.001, CFI = 0.93, TLI = 0.92, RMSEA = 0.05 (CI = 0.04, 0.06), SRMR = 0.03] and sub-elite athletes [χ2(436) = 974.87, *p* < 0.001, CFI = 0.94, TLI = 0.94, RMSEA = 0.05 (CI = 0.04, 0.05), SRMR = 0.02] responses, indicated a satisfactory fit to the data (Hair et al., 2014). All items showed moderate to strong factor loadings ranging from 0.626 to 0.871 (elite) and 0.621 to 0.873 (sub-elite) (see Table 3).

### Structural Model

Overall model fit for structural models was found to be satisfactory for both elite [χ2(470) = 1438.58, *p* < 0.001, CFI = 0.92, TLI = 0.91, RMSEA = 0.05 (CI = 0.05, 0.06), SRMR = 0.05] and sub-elite [χ2(470) = 1174.87, *p* < 0.001, CFI = 0.93, TLI = 0.93, RMSEA = 0.06 (CI = 0.05, 0.06), SRMR = 0.04] subsamples.

An examination of the path coefficients for each model in Table 4, identified several different relationships between the groups. Athletes’ perceptions of parents’ encouragement and reinforcement showed significant relationships on athletes’ performance level in both models (*p* < 0.01; H1a and H1b), whereas the relationships between parental modeling and performance level were significant for the elite group (β = 0.15, *p* < 0.01), but not significant for the sub-elite group (*p* > 0.05) – H1d. In contrast, perceptions of parental instruction were not significantly associated with performance for both groups (*p* > 0.05) – H1c. Perceptions of parental encouragement showed a significant positive effect on intrinsic motivation, self-efficacy, and self-regulation for both elite and sub-elite groups (*p* < 0.05) – H21a, H21b, and H21d. The path estimates of perceptions of parents’ encouragement and social efficacy with coaches were not significant for both groups (*p* < 0.05) – H21c. Perceptions of parents’ reinforcement were significantly associated with intrinsic motivation, self-efficacy and self-regulation for both groups (*p* < 0.05) – H22a, H22b, and H22d – while not significant with social efficacy with coach for both groups (*p* > 0.05) – H22c. Furthermore, perceptions of parents’ instruction were negatively linked with intrinsic motivation and social efficacy with coach for both groups (*p* < 0.05; H23a and H23c). Moreover, perceptions of parents’ modeling were related with performance for the elite group (*p* < 0.05). In contrast, perceptions of parents’ modeling were significant with self-efficacy (β = 0.09, *p* < 0.01) and social efficacy (β = 0.12, *p* < 0.01) for the sub-elite group – H24a, H24b, H24c, and H22d. For the relationships between psychological variables and performance, results showed significant associations between self-efficacy, self-regulation, social efficacy with coach, and intrinsic motivation with performance for both groups (*p* < 0.05) – H3a, H3b, H3c, and H3d. Together, perceptions of parenting practices and proposed psychological variables explained approximately 28% of the variance for elite (*R*^2^ = 0.28) and 20% for the sub-elite group (*R*^2^ = 0.20).

**TABLE 4:** Summary results of the structural model for each of the subsamples.

**Path**			**Elite**		**Sub-elite**		**Power (1 – β)**
		**Confirmed?**	***β***	**CR**	***β***	**CR**	
H1a	Encouragement → Achievement	Yes	0.48**	15.62	0.36**	13.34	0.93
H1b	Reinforcement → Achievement	Yes	0.22**	8.01	0.24**	8.76	0.14
H1c	Instruction → Achievement	No	–0.02	–1.37	–0.02	–1.31	0.05
H1d	Modeling → Achievement	Partially	0.15**	5.55	0.05	1.86	0.99
H21a	Encouragement → Intrinsic mot.	Yes	0.52**	22.45	0.41**	16.30	0.88
H21b	Encouragement → Self-efficacy	Yes	0.10**	4.26	0.09**	3.66	0.12
H21c	Encouragement → Social efficacy	No	0.02	1.18	0.01	0.98	0.34
H21d	Encouragement → Self-regulation	Yes	0.11**	4.55	0.09**	3.61	0.23
H22a	Reinforcement → Intrinsic mot.	Yes	0.12**	4.91	0.12**	4.90	0.05
H22b	Reinforcement → Self-efficacy	Yes	0.10**	4.13	0.13**	5.04	0.30
H22c	Reinforcement → Social efficacy	No	0.02	1.03	0.01	0.15	0.34
H22d	Reinforcement → Self-regulation	Yes	0.09**	3.61	0.10**	3.23	0.11
H23a	Instruction → Intrinsic mot.	Yes	–0.09**	3.65	–0.14**	5.44	0.61
H23b	Instruction → Self-efficacy	No	–0.01	–1.12	–0.02	–1.34	0.21
H23c	Instruction → Social efficacy	Yes	–0.12**	4.89	−0.06*	1.78	0.86
H23d	Instruction → Self-regulation	No	0.01	1.11	0.02	1.32	0.21
H24a	Modeling → Intrinsic mot.	Partially	0.21**	7.89	0.02	1.30	1.00
H24b	Modeling → Self-efficacy	Yes	0.10**	4.25	0.09**	3.58	0.12
H24c	Modeling → Social efficacy	Yes	0.08**	3.15	0.12**	4.90	0.49
H24d	Modeling → Self-regulation	Partially	0.16**	5.25	0.02	1.29	0.99
H3a	Intrinsic mot. → Achievement	Yes	0.34**	11.14	0.29**	10.05	0.40
H3b	Self-efficacy → Achievement	Yes	0.28**	9.75	0.27**	9.14	0.09
H3c	Social efficacy → Achievement	Yes	0.15**	5.81	0.18**	6.76	0.25
H3d	Self-regulation → Achievement	Yes	0.36**	12.03	0.26**	9.10	0.86

*p < 0.05, **p < 0.01; CR = critical ratio.

Following Byrne’s (2010) suggestions, a multigroup CFA was performed to analyze whether the path coefficients differed significantly between elite and sub-elite groups. The fit of the unconstrained model [χ2(940) = 1823.29, *p* < 0.001, CFI = 0.934, TLI = 0.931, RMSEA = 0.039 (CI = 0.038, 0.042), SRMR = 0.04] was satisfactory. As well as for the constrained measurement weights model [χ2(946) = 1900.81, *p* < 0.001, CFI = 0.930, TLI = 0.922, RMSEA = 0.040 (CI = 0.037, 0.041), SRMR = 0.05], and constrained structural weights [χ2(952) = 1936.21, *p* < 0.001, CFI = 0.922, TLI = 0.913, RMSEA = 0.051 (CI = 0.048, 0.055), SRMR = 0.05] models. The χ^2^ statistic showed significant differences between unconstrained and constrained measurement weights models [Δχ2(44) = 77.52, *p* = 0.001], and between unconstrained and constrained structural weights models [Δχ2(64) = 111.92, *p* = 0.001].

**FIGURE 2:**
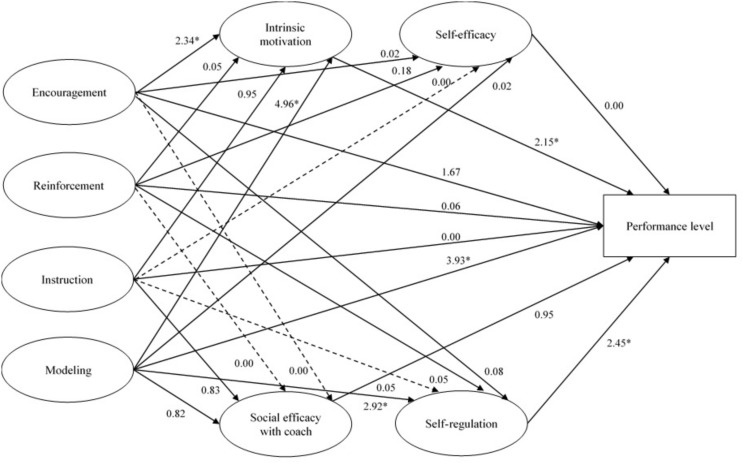
Critical ratios (CR) for differences in structural parameters between both subsamples. ^*^CR > 1.96, *P* < 0.05. The non-significant paths for both subsamples are presented by dashed lines.

An inspection to critical ratios (CR) for differences between parameters revealed that six structural paths differ significantly between groups (CR > 1.96, *p* < 0.05). Specifically, the path between perceptions of parental encouragement and intrinsic motivation showed differences among groups (CR = 2.34, *p* < 0.05). Moreover, perceptions of parental modeling revealed a significantly different relationship on performance level (CR = 3.93, *p* < 0.05), on intrinsic motivation (CR = 4.16, *p* < 0.05), and on self-regulation (CR = 2.92, *p* < 0.05). In addition, the relationships among intrinsic motivation (CR = 2.15, *p* < 0.05) and self-regulation (CR = 2.45, *p* < 0.05) with performance were significantly different between elite and sub-elite youth athletes. The path model presented in Figure 2 shows the summary of differences within the models for both elite and sub-elite groups.

To verify the magnitude of the differences between elite and sub-elite proportions (p1 – p2), a *post hoc* power analysis was completed using GPower 3.1 (Faul et al., 2007). The significance level of *p* < 0.05 was adopted for the suitable level of greater than 0.80. *Post hoc* analyses showed in Table 4 revealed statistical power for differences between groups.

## Discussion

In the present study, we examined the simultaneous associations between athlete’s perceptions of parenting practices, self-reported psychological variables, and youth athletes’ level of performance in sport. The data supported adequacy of the parental involvement in sport model in explaining how parents’ behaviors are associated with their children’s achievement of performance via important psychological variables.

### Perceptions of Parents’ Behaviors and Level of Performance in Sport

According to the model of parental involvement in sport, young athletes’ perceptions of parents’ behaviors are related to their sport participation (Teques et al., 2018). The findings of this study suggest that perceived encouragement and reinforcement were positively associated with both elite and sub-elite athletes. These results are in accordance with existing literature concerning family influences on children’s development in sport (Côté, 1999; Wolfenden and Holt, 2005; Knight et al., 2016). Parents are providers of emotional support through encouragement and reinforcement behaviors and show commitment to doing one’s best as values of achievement.

The relationship between parental modeling and performance level was significantly different between groups. Findings of this study showed that modeling as a parental role was only linked to elite athletes. In line with Bloom (1985), parents’ commitment to doing one’s best is an important value they taught their children; additionally, parental efforts in modeling hard work and setting high standards of performance was recognized by children. Although modeling is often cited conceptually as important for child’s values, attitudes and behavior (Bandura, 1997), empirical studies relating parental modeling to achievement of a high standard in sport are rare. The current study examined athletes’ perceptions of parental behaviors as values of hard work to impact young athletes’ development in sport. Scholars used self-report questionnaires on parents’ role modeling designed to assess participants’ appraisals of the extent of their parents’ involvement in sport and physical activity, revealing inconsistent findings. Babkes and Weiss (1999), for example, found that the perceptions of competence, intrinsic motivation and enjoyment of young soccer players were related with their appraisals of parents’ as a positive role model in physical activity. Differing results were found by Fredricks and Eccles (2005) who reported that parents’ role modeling, defined as time participation in sport activities, were not a determinant of children’s sport participation. Even though modeling likely plays a role in the achievement of a high standard in sport, additional research is needed before its role can be fully understood.

### Perceptions of Parents’ Behaviors and Athletes’ Psychological Variables

A consistent finding across both elite and sub-elite groups was the relationships between perceptions of parents’ encouragement and reinforcement, and important psychological variables in sport, such as self-efficacy, self-regulation, and intrinsic motivation. The importance of parental emotional support is consistently evidenced to better understand how athletes achieve elite performance in youth sport (Côté, 1999; Holt and Dunn, 2004; Wolfenden and Holt, 2005). As well as Ward et al. (2007) suggested that elite players mentioned their parents as source of support and encouragement. However, previous research presented parents’ behaviors using a descriptive view of emotional support, and the current findings point in evidence the ways in which sport parents shows adequate emotional support and how this optimize their child’s psychological variables in sport. Specifically, results of the current study extend previous knowledge suggesting that the linkage between higher amounts of perceived parental encouragement and reinforcement and levels of performance could be explained by stronger beliefs of young athletes in their ability to master their sport activities and higher interest in sport practice for the sense of accomplishment derived from trying to surpass oneself.

The negative association between parental instructional behavior and young athletes’ intrinsic motivation to accomplish and social efficacy for relating with the coach may suggest that parental instruction provided to directly command action may undermine athletes’ sport experience. As suggested by Holt et al. (2008), this type of parental instruction represents 35% of the recorded comments during games, which means that over one third of the parents’ comments may have the potential for undermining athletes’ intrinsic motivation. From a self-determination perspective, performance pressure based on excessive instructions or using controlling words such as “should,” are particularly insidious to motivation quality (Ryan and Deci, 2006). Furthermore, the negative relationship between perceptions of parent’s instructional behaviors and the confidence to be socially related with the coach reinforce the idea that the perceptions of parental instruction by young athletes could undermine the quality of the coach-athlete relationship (Jowett and Timson-Katchis, 2005). In fact, demanding parents displayed behaviors that were generally disliked by the coach and the athlete, such as providing technical and tactical advice (Averill and Power, 1995).

### Psychological Variables and Level of Performance in Sport

In line with the model of parental involvement in sport, self-efficacy, social self-efficacy with the coach, intrinsic motivation and strategies for self-regulation are associated with athletes’ achievement of elite performance in sport. In this respect, findings of this study revealed a significant linkage between perceptions of parental modeling, self-efficacy and social self-efficacy for relating to coaches, and performance level. Previous research highlighted differences between elite and sub-elite players on self-efficacy beliefs in their sport experiences (Mallett and Hanrahan, 2004). However, the present study extends our understanding by suggesting that the relationship of achievement with perceiving parents as models of hard work could be explained by athletes’ stronger beliefs in their ability to master their sport activities. These results reinforce the view that self-perceptions of efficacy operate as cognitive mediators of action (Bandura, 1997). Also, the present results corroborate research in academic context that found that students’ efficacy to relate effectively with their teachers and peers is associated with math achievement (Patrick et al., 2007). Further clarification of the relative importance of self-efficacy and social efficacy beliefs in relation to achievement or other motivational outcomes seems merited in sport.

### Limitations and Future Directions

Although the current study contributes to a broader understanding of parenting practices in the achievement of elite performance in sport, several limitations are worth mentioning. First, data were cross sectional, which limits causal interpretations of the regression effects. Although the hypothesized associations described in the structural model demonstrate an explanation that fits with the data, longitudinal studies should be developed to assess reciprocal effects to enhance the understanding of how parenting practices, psychological constructs, and elite youth achievement reciprocally impact each other across athletes’ developmental stages (Côté, 1999). Second, it seems clear from the levels of variance explained by the model that there are other factors implicated in youth elite achievement in sport. Additional research is warranted to better understand how parenting practices relates to other specific sport performance characteristics. For example, parenting styles seem to indirectly influence on young athlete’s behaviors, while parental practices have a direct effect on young athlete’s behavior (Holt et al., 2009). Also, various personality characteristics have been associated with talented soccer players, including self-concept, fear of failure, hope for success or self-optimism (Feichtinger and Höner, 2015), and expectancies for success have long been recognized as important variables to explain achievement behaviors, such as task persistence and task choice (Fredricks and Eccles, 2004). Third, it should be recognized that findings of this study might differ depending on adolescent’s gender (Fredricks and Eccles, 2005). Future studies are thus needed to further explore the structural mean differences between these two groups. Fourth, the scales used to evaluate psychological constructs (i.e., self-efficacy, social efficacy to relate with the coach, intrinsic motivation, and self-regulatory strategies) were purposely validated for this study. For this reason, we decided to perform a CFA and these scales showed relevant psychometric characteristics, including item individual reliability, scale composite reliability, factorial validity, and convergent and discriminant validity. However, due to the importance shown by these psychological constructs in this study, researchers should validate these instruments in full. Fifth, modeling and self-efficacy demonstrated problems of convergent validity, evidenced by low to moderate correlations between variables. Problems with convergent validity may be due to the fact that the constructs are composed by too few indicators (Kline, 2011). Future studies may explore the functioning of these scales in relation to other psychological constructs. Finally, participants are from a western European country, widely held by young athletes from two-parent families of middle-class status in order to examine parenting practices in youth elite sport. Most of the research on parental involvement has used similar samples. An important area of future research is how parents from different types of families (e.g., single-parent) with fewer resources support children’s competitive sport. For example, different relationships between parental practices and youth elite achievement may be obtained with athletes from different cultures and socioeconomic status (cf., Holt and Dunn, 2004; Moraes et al., 2004).

## Conclusion

In conclusion, the model of parental involvement since its development has contributed to an integrative rationale for research on youth sport, examining the relations between parent-child relationship and child psychological outcomes (Teques et al., 2015, 2018). Based on this line of research, the current study also contributes to expand knowledge about how parents’ behaviors are associated with their children’s achievement of elite performance in sport via important psychological variables.

## Data Availability

The datasets for this manuscript are not publicly available because the datasets generated during and/or analyzed during the current study are available from the corresponding author on reasonable request. Requests to access the datasets should be directed to pteques@ipmaia.pt.

## Ethics Statement

This study was carried out in accordance with the recommendations of the Ethical Committee of the Faculty of Human Kinetics with written informed consent from all subjects. All subjects gave written informed consent in accordance with the Declaration of Helsinki. The protocol was approved by the Ethical Committee of the Faculty of Human Kinetics.

## Author Contributions

PT and SS were enrolled in the study design, data collection, and wrote the first draft of the manuscript. PT, AR, and CS participated in the data analysis and wrote the methodology and results. PT, SS, and LC participated in the final revisions of the manuscript. All authors read and approved the final version of the manuscript and agreed with the order of the presentation of the authors.

## Conflict of Interest Statement

The authors declare that the research was conducted in the absence of any commercial or financial relationships that could be construed as a potential conflict of interest.
